# Safety of metformin continuation in diabetic patients undergoing invasive coronary angiography: the NO-STOP single arm trial

**DOI:** 10.1186/s12933-023-01744-4

**Published:** 2023-02-06

**Authors:** Mauro Chiarito, Jorge Sanz-Sanchez, Raffaele Piccolo, Francesco Condello, Gaetano Liccardo, Matteo Maurina, Marisa Avvedimento, Damiano Regazzoli, Paolo Pagnotta, Hector M. Garcia-Garcia, Roxana Mehran, Massimo Federici, Gianluigi Condorelli, Jose Luis Diez Gil, Bernhard Reimers, Giuseppe Ferrante, Giulio Stefanini

**Affiliations:** 1 Cardio Center, IRCCS Humanitas Research Hospital, Rozzano, Milan, Italy; 2 Department of Biomedical Sciences, Humanitas University, Pieve Emanuele, Milan, Italy; 3 Hospital Universitario y Politécnico La Fe, Valencia, Spain; 4 Centro de Investigación Biomedica en Red, Madrid, Spain; 5 Department of Advanced Biomedical Sciences, University of Naples Federico II, Naples, Italy; 6 Section of Interventional Cardiology, MedStar Washington Hospital Center, Washington, DC USA; 7 Icahn School of Medicine at Mount Sinai, New York City, NY USA; 8 Department of Systems Medicine, University of Rome Tor Vergata, Rome, Italy; 9 Center for Atherosclerosis, Policlinico Tor Vergata, Rome, Italy

**Keywords:** Metformin, Metformin-associated lactic acidosis, Percutaneous coronary intervention, Coronary angiography

## Abstract

**Background:**

Despite paucity of data, it is common practice to discontinue metformin before invasive coronary angiography due to an alleged risk of Metformin-Associated Lactic Acidosis (M-ALA). We aimed at assessing the safety of metformin continuation in diabetic patients undergoing coronary angiography in terms of significant increase in lactate levels.

**Methods:**

In this open-label, prospective, multicentre, single-arm trial, all diabetic patients undergoing coronary angiography with or without percutaneous coronary intervention at 3 European centers were screened for enrolment. The primary endpoint was the increase in lactate levels from preprocedural levels at 72-h after the procedure. Secondary endpoints included contrast associated-acute kidney injury (CA-AKI), M-ALA, and all-cause mortality.

**Results:**

142 diabetic patients on metformin therapy were included. Median preprocedural lactate level was 1.8 mmol/l [interquartile range (IQR) 1.3–2.3]. Lactate levels at 72 h after coronary angiography were 1.7 mmol/l (IQR 1.3–2.3), with no significant differences as compared to preprocedural levels (p = 0.91; median difference = 0; IQR − 0.5 to 0.4 mmol/l). One patient had 72-h levels ≥ 5 mmol/l (5.3 mmol/l), but no cases of M-ALA were reported. CA-AKI occurred in 9 patients (6.1%) and median serum creatinine and estimated glomerular filtration rate remained similar throughout the periprocedural period. At a median follow-up of 90 days (43–150), no patients required hemodialysis and 2 patients died due to non-cardiac causes.

**Conclusions:**

In diabetic patients undergoing invasive coronary angiography, metformin continuation throughout the periprocedural period does not increase lactate levels and was not associated with any decline in renal function.

*Trial registration*: The study was registered at Clinicaltrials.gov (NCT04766008).

**Graphical Abstract:**

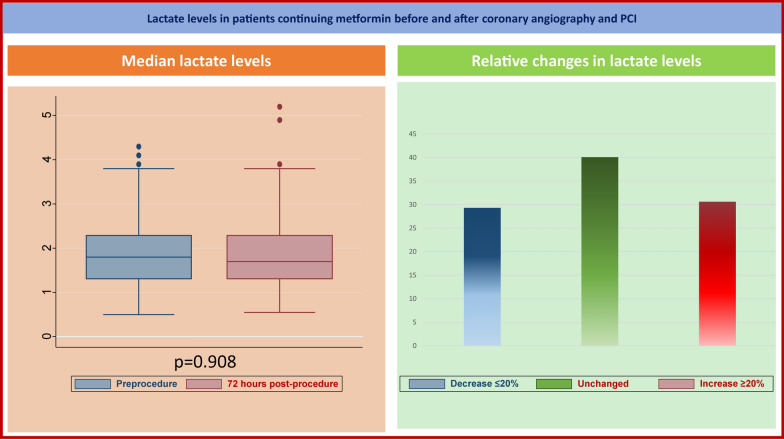

**Supplementary Information:**

The online version contains supplementary material available at 10.1186/s12933-023-01744-4.

## Background

In the past decades, the prevalence of diabetes mellitus (DM) has significantly increased worldwide, and it is estimated that 592 million people will be affected by DM in 2035 [1]. DM is extremely common among patients with coronary artery disease, with prevalence reaching up to 40% in cohort of patients undergoing percutaneous coronary intervention (PCI) [2]. Metformin is the most common hypoglycemic drug prescribed worldwide in diabetic patients [3], although its use is potentially hampered by the risk of Metformin-Associated Lactic Acidosis (M-ALA), usually limited to patients at increased risk of metformin accumulation [4]. Despite paucity of robust clinical evidence, metformin is commonly discontinued on the day of coronary angiography and for the following 48 h [5], as it is largely eliminated via renal excretion and its plasma half-life is 4 to 8.7 h in patients with preserved renal function [6]. The 2018 European Society of Cardiology Guidelines on myocardial revascularization suggest to withhold metformin only in patients with deteriorated renal function (Class of recommendation I, level of evidence C) [7]. As a result, the safety of metformin continuation in patients undergoing coronary angiography and PCI remains a matter of debate, with lack of an universal agreement regarding the need to discontinue it, leading to great heterogeneity in daily clinical practice [8]. Indeed, the clinical relevance of M-ALA in patients undergoing coronary angiography has recently come into question [9]. Conversely, the discontinuation of metformin could imply delay in coronary angiography and result in poor glycemic control, which in turn increases the risk of cardiovascular events and contrast-associated acute kidney injury (CA-AKI) [10]. A number of observational and randomized studies comparing metformin continuation versus interruption before and following coronary angiography/PCI reported no differences in M-ALA, CA-AKI, serum lactate and bicarbonate levels, or short-term mortality [11, 12]. However, these studies were not powered to detect a clinically relevant difference in the risk of M-ALA or CA-AKI and did not report preprocedural lactate levels. Therefore, we designed the present study to assess whether metformin continuation in diabetic patients undergoing coronary angiography with or without PCI is associated with an increase of lactate levels throughout the periprocedural period.

## Research design and methods

### Study design and population

This is an open-label, prospective, multicentre, single-arm trial. All diabetic patients undergoing coronary angiography with or without PCI at 3 participating centers (Additional file 1: Table S1) between January 11th, 2020, and March 3rd, 2022, were screened for enrolment. The CONsolidated Standards of Reporting Trials (CONSORT) checklist is reported in the Additional file 1: Appendix. Inclusion criteria were broad and included all diabetic patients treated with metformin, irrespective of concomitant hypoglycemic medications. Patients with severely impaired left ventricular ejection fraction (LVEF) < 35%, moderate to severe impairment of renal function, defined as an estimated glomerular filtration rate (eGFR) < 45 ml/min/1.73 m^2^, advanced liver disease (Child–Pugh B or C), with severe to very severe chronic obstructive pulmonary disease (GOLD class 3 to 4), those with known coronary anatomy scheduled for elective PCI with high probability of large amount of contrast use [calculated as 3*estimated glomerular filtration rate (eGFR)] [13], undergoing primary PCI, and those scheduled for cardiac surgery in the following 5 days were excluded. All patients underwent blood gas analysis reporting lactate levels and venous blood sample to evaluate glycemia, complete blood cell count, renal and liver function within 24 h before coronary angiography. Lactate and creatinine measurement were assessed at 72 h after coronary angiography, whereas a complete blood gas analysis was mandatory in case of lactate levels above 3 mmol/l. All patients with any deviation from the study protocol (e.g., lack of lactate levels after coronary angiography) were prospectively followed and enrolled in a parallel registry.

Coronary angiography and PCI were performed according to standard techniques; stent and technique choice were based on operators’ preferences and there were no related exclusions. A low-osmolar, non-ionic contrast medium (iomeprol) has been used during the entire study period. Per the standard clinical protocol, up to 1 ml/Kg/h of saline up to 12 h before and up to 24 h after PCI were strongly recommended, although not mandatory. The total amount and type of hydration infused were prospectively collected. Antithrombotic regimen was based on parenteral heparin, and the use of oral aspirin plus a P2Y_12_ inhibitor, as well as therapy with statins, were based on patients’ clinical presentation and treating physicians’ preference.

A dedicated data coordinating center performed all data management. All data were entered prospectively in the database.

The study complies with the Declaration of Helsinki and each patient provided written informed consent before coronary angiography. The study was approved by the Institutional Review Board at each site before enrollment and is registered with ClinicalTrials.gov, number NCT04766008.

### Endpoint definitions and follow-up

The primary endpoint was the difference in lactate levels from preprocedural levels at 72 h after coronary angiography. Secondary endpoints included CA-AKI, M-ALA, and all-cause mortality. CA-AKI was defined according to the Acute Kidney Injury Network criteria as an increase by ≥ 50% or ≥ 0.3 mg/dl within 72 h after coronary angiography compared to pre-coronary angiography serum creatinine [14]. M-ALA was defined as a blood pH < 7.35 with concomitant increase in lactate levels (> 5.0 mmol/l).[4] Chronic kidney disease (CKD) was defined as an eGFR of ≤ 60 ml/min/1.73 m^2^, per the Modification of Diet in Renal Disease formula [15, 16]. Myocardial infarction was defined according to the IV Universal Definition of myocardial infarction [17]. Definite stent thrombosis and definite/probable stent thrombosis were defined according to the Academic Research Consortium 2 criteria [18]. The definition of cardiovascular death included any death because of an immediate cardiovascular cause. Bleeding events were classified according to the Bleeding Academic Research Consortium definition [19]. Clinical follow-up was performed in-hospital, at 3 days and at least after 30 days from coronary angiography with telephone or office visits.

### Statistical analysis

The sample size was calculated to detect with 90% power a prespecified increase of lactate of 20% from preprocedural levels, with a "one-side" probability of alpha error of 0.025. Sample size calculation was based on data from our historical cohort of diabetic patients taking metformin scheduled for coronary angiography, presenting a mean value of lactate before coronary angiography of 1.2 ± 0.7 mmol/l. A total of 150 patients was planned to be enrolled assuming dropout and attrition rate of 20% and 5%, respectively.

Continuous variables were reported as mean ± standard deviation or median and interquartile range (IQR) and compared with Student’s t test or Mann–Whitney or Wilcoxon tests on the basis of normality of data, verified by Shapiro–Wilk test. Categorical variables are reported as N (%) and were compared with χ^2^-test with Yates’ correction for continuity or the Fisher exact test, as appropriate. Subgroup analyses were performed to assess differences in the following prespecified subgroups: patients taking other hypoglycemic drugs in addition to metformin, patients undergoing PCI, patients with mild to moderate renal function impairment (eGFR < 90 and > 60 ml/min). Furthermore, linear regression was performed to assess the correlation between clinical and laboratory variables with absolute lactate levels variation between preprocedural and 72-h levels. Two-sided p levels < 0.05 were considered statistically significant. Statistical analyses were performed using Stata, version 16.0 (StataCorp LLC).

## Results

### Baseline features

**Fig. 1:**
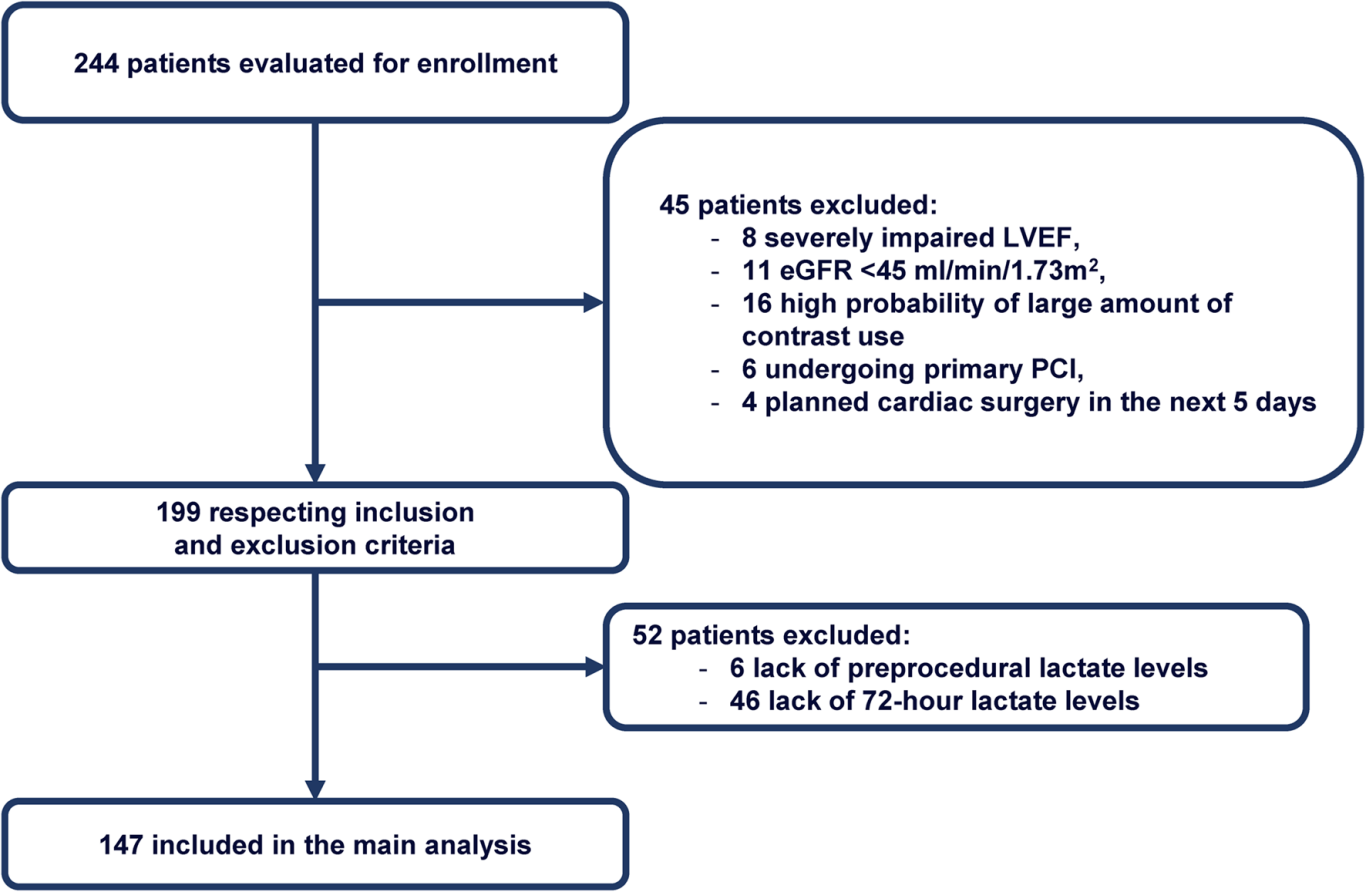
Study flow chart. *eGFR* estimated glomerular filtration rate, *LVEF* left ventricular ejection fraction, *PCI* percutaneous coronary intervention

Figure 1 shows the study flow chart: between January 11th, 2020, and March 3rd, 2022, a total of 199 patients were deemed eligible for enrolment; 6 and 46 patients were excluded due to the lack of lactate levels before coronary angiography and at 72 h after the procedure, respectively, resulting in a total of 147 patients included in the main analysis. Baseline clinical, laboratory, and procedural features are reported in Table 1.

**Table 1:** Baseline characteristics of the included population

Baseline clinical characteristics	Study population (n = 147)
Age, years	71 (65–77)
Female	28 (19)
Body mass index, kg/m^2^	27.7 (25.8–31.8)
Hypertension	133 (91)
Dyslipidemia	123 (83.7)
Chronic kidney disease	21 (14.3)
Peripheral artery disease	31 (21.1)
Atrial fibrillation	20 (13.6)
Moderate COPD	6 (4.1)
Mild to moderate liver disease	3 (2)
Anemia	39 (26.5)
History of smoking	74 (50.3)
Current smoker	43 (29.3)
Previous myocardial infarction	39 (26.5)
Previous CABG	21 (14.3)
Previous PCI	63 (42.9)
Previous stroke	14 (9.5)
LVEF, %	55 (52–60)
Clinical presentation	
Silent ischemia	53 (36.1)
Stable angina	67 (45.6.9)
Unstable angina	8 (5.4)
NSTEMI	10 (6.8)
Planned cardiac surgery	7 (4.8)
NYHA classification	
I	77 (54.6)
II	50 (35.5)
III	14 (9.9)
Glycated Hemoglobin, %	6.8 (6.3–7.7)
Medical treatment	
Metformin daily dosage, mg	1700 (1000–2000)
Hypoglycemic therapy	
Insulin	23 (15.6)
SGLT2 inhibitors	32 (21.8)
GLP1 analogues	14 (9.5)
DPP-4 inhibitors	28 (19.1)
Sulphonylureas	15 (10.2)
Thiazolindiones	7 (4.7)
Statins	123 (83.7)
High-potency and high dosage statin	59 (40.1)
ACE inhibitors or ARB	113 (76.9)
MRAs	19 (12.9)
Loop diuretics	27 (18.4)
Thiazide diuretics	28 (19.1)
Procedural features	
Access	
Transradial	132 (89.8)
Femoral	13 (8.8)
Brachial	1 (0.7)
Ulnar	1 (0.7)
PCI	85 (57.8)
Bypass angiography	7 (4.8)
Contrast media amount, ml	90 (50–135)
Hydration	
Pre-procedural hydration	93 (63.3)
Pre-procedural hydration amount, ml	160 (0–500)
Post-procedural hydration	127 (86.4)
Post-procedural hydration amount, ml	500 (250–1000)

*ACE* angiotensin converting enzyme, *ARB* angiotensin receptor blocker, *CABG* coronary aortic bypass graft, *COPD* chronic obstructive pulmonary disease, *DPP-4* dypeptil-peptidase 4, *MRA* mineralcorticoid receptor antagonist, *PCI* percutaneous coronary intervention, *SGLT-2*: sodium glucose cotrasporter

Most included patients were male (n: 119, 81%), and median age was 71 years (65–77). Only 23 patients (15.6%) presented with type 2 insulin dependent DM, median HbA1C was 6.8% (6.3–7.7), and preprocedural glycemia was 131 (114–154) mg/dL. CKD was present in 21 patients (14.3%), and anemia in 39 patients (26.5%). A total of 18 (12.2%) patients underwent coronary angiography due to acute coronary syndrome (8 for unstable angina and 10 for non-ST elevation myocardial infarction) and median LVEF was 55% (52–60). According to the Mehran risk score [20], 73 patients (49.7%) were deemed to be at low risk for CA-AKI, while 47 (32%), 19 (12.9%) and 8 (5.4%) resulted to be at moderate, high, and very high risk for CA-AKI, respectively. An improved stratification was observed using the Mehran 2.0 risk score [10], which classified only 4 patients at high and 1 patient at very high risk for CA-AKI (Additional file 1: Table S2).

Median metformin dose was 1700 (1000–2000) mg daily, and half of the population (71 patients, 48.3%) was treated with additional hypoglycemic drugs, including a total of 32 patients (21.8%) treated with sodium-glucose co-transporter 2 inhibitors. Renin–angiotensin–aldosterone system inhibitors were largely used in the study population: angiotensin-converting enzyme inhibitors or angiotensin receptor blockers were assumed by 113 (76.9%) patients, and mineralcorticoid receptor antagonist by 19 (12.9%) patients. Statins were used in most patients (n: 123, 83.7%): more in details, 59 patients (40.1%) assumed high-potency statin at high dosage. Preprocedural hydration was administered in 93 patients (63.3%), for a median total amount of 160 (0–500) ml. Bicarbonate and n-acetylcysteine were used only in 9 patients.

### Procedural features

Most patients underwent transradial coronary angiography (n: 132, 89.8%), while brachial and femoral access were used in 1 (0.7%) and 13 (8.8%) patients, respectively. Median contrast volume was 90 (50–135) ml. PCI was performed in 85 patients (57.8%). Procedural adverse events were rare: one patient experienced coronary perforation, treated with coil implantation; and one patient had a periprocedural myocardial infarction. Post-procedural hydration was administered to 127 patients (86.4%), for a median amount of 500 (250–1000) ml. Adequate glycemic control was observed before and after the procedure (Table 2).

**Table 2:** Median levels of laboratory values and eGFR before and at 72-h after coronary angiography and PCI

	Pre-procedural, median (IQR)	72-h, median (IQR)
Lactate, mmol/l	1.8 (1.3–2.3)	1.7 (1.3–2.3)
Creatinine, mg/dl	0.89 (0.76–1.07)	0.89 (0.77–1.11)
eGFR, ml/min/1.73 m^2^	79 (65–97)	79 (62–96)
Urea, mg/dl	38 (33–48)	39 (31–48)
Glycemia, mg/dl	131 (114–154)	132 (113–154)

*eGFR* estimated glomerular filtration rate, *IQR* interquartile range

## Outcomes

### Postprocedural lactate levels

**Fig. 2:**
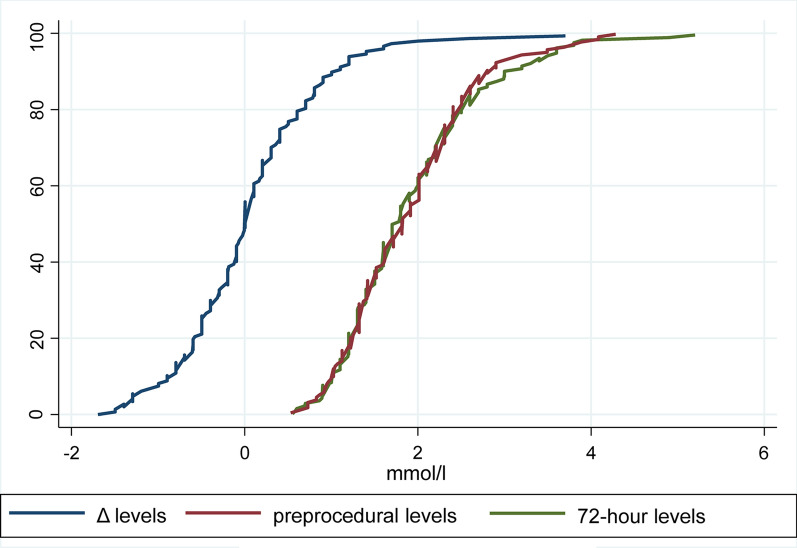
Cumulative curves reporting preprocedural and 72-h lactate levels and their difference. Cumulative curves depict the sum of the frequency of preprocedural, 72-h lactate levels and their difference for all patients

Median preprocedural lactate level was 1.8 (1.3–2.3) mmol/l, with increased level reported in patients treated with higher dosage of metformin (ρ = 0.18, p = 0.027), while there was no correlation with eGFR (p = 0.984). Lactate levels measured at 72 h after coronary angiography were 1.7 (1.3–2.3) mmol/l, with no significant differences compared to preprocedural levels (p = 0.908), as the median difference between pre- and postprocedural levels was 0 (− 0.5 to 0.4) mmol/l (Fig. 2). Nonetheless, 45 patients (30.6%) had an increase of 20% as compared with the baseline value and 14 patients (9.5%) had lactate levels ≥ 3 mmol/l, while only one patient had levels ≥ 5 mmol/l (5.3 mmol/l; Graphical abstract). No cases of M-ALA were reported. We did not observe any impact on the difference between preprocedural and 72-h lactate levels of concomitant hypoglycemic drugs on top of metformin, PCI after coronary angiography, and mild to moderate renal function impairment. No differences in postprocedural or absolute differences between pre- and postprocedural lactate level were observed between patients receiving or not preprocedural hydration (p = 0.150 and p = 0.176, respectively). As reported in Additional file 1: Table S3, the preprocedural, postprocedural and total amount of hydration were not significantly correlated to postprocedural or absolute differences between pre- and postprocedural lactate level.

### Clinical outcomes

As reported in Table 3, CA-AKI was reported only in 9 patients (6.1%, Stage 1 according to the AKIN classification in all cases), and its occurrence did not correlate with metformin dosage, absolute differences between pre- and postprocedural level of lactate, and absolute level of postprocedural lactate. None of the patients who developed CA-AKI required hemodialysis or died in-hospital.

**Table 3:** In-hospital and mid-term clinical outcome

Clinical outcome	N (%)
M-ALA	0 (0)
CA-AKI	9 (6.1)
All-cause death	2 (1.4)
In-hospital death	0 (0)
Cardiac death	0 (0)
MI	1 (0.7)
Definite or probable ST	1 (0.7)
Any revascularization	5 (3.5)
Stroke	2 (1.5)
Any rehospitalization	14 (9.5)
Any bleeding	5 (3.4)

*CA-AKI* contrast-associated kidney injury, *M-ALA* metformin-associated lactic acidosis, *MI* myocardial infarction, *ST* stent thrombosis

Similarly, at a median follow-up of 90 days (43–150), there were no patients requiring hemodialysis and only 2 patients died, due to non-cardiac causes (related to respiratory insufficiency, secondary to coronavirus disease 19, and to septic shock). A total of 14 patients required rehospitalization (only in 3 cases for cardiovascular reasons).

## Conclusions

Metformin is recommended as first line therapy in patients with type 2 DM and is one of the most common drugs assumed by patients scheduled for coronary angiography. The present study expands the limited evidence on the safety of metformin continuation in patients undergoing coronary angiography and PCI. The main findings of our study are as follows:

- In diabetic patients without high-risk features for metformin accumulation, metformin continuation before and after coronary angiography with or without PCI resulted in similar preprocedural and 72-h lactate levels.
- The risk of CA-AKI was low, and no patients required hemodialysis during the index hospitalization or at mid-term follow-up.

### Glycemic control and adverse events in patients undergoing coronary angiography and PCI

Early guidelines recommendations on the discontinuation of metformin before coronary angiography were based on expert opinion, due to the paucity of evidence in this setting. A common recommendation was to hold metformin on the day of contrast media administration and for the following 48 h, irrespective of the risk of CA-AKI and metformin accumulation [5, 8, 21, 22]. Indeed, this rule-of-thumb is based on the risk of developing CA-AKI after PCI among diabetic patients, which could in turn result in metformin accumulation and subsequent M-ALA, encumbered by an estimated mortality reaching up to 50%. Metformin is commonly replaced by insulin or no hypoglycemic therapy before and after the procedure [23]. Of note, this practice could lead to hyperglycemia, which in turn has been consistently associated with an increased risk of CA-AKI [24], even in absence of diabetes, as it might induce medullary hypoxia and tubular cells injury, secondary to reduced nitric oxide availability and increased oxidative stress [10, 25, 26] The deleterious effect of hyperglycemia in this setting was confirmed by Shah et al., who randomized 172 diabetic patients to continue versus hold glucose-lowering medications before coronary angiography. The authors reported that the better glycemic control achieved in the continue group than in the hold group resulted in a significantly lower risk of reduction in eGFR after coronary angiography in the group that continued metformin as compared with who discontinued metformin. Importantly, this finding further highlights that metformin is not directly nephrotoxic. Moreover, the continue group had reduced platelet activity, suggesting a further potential beneficial effect of glycemic control in this setting [27, 28].

### Risk of CA-AKI and M-ALA with metformin continuation before and after coronary angiography and PCI

Based on this evidence, the risk–benefit trade-off for metformin continuation or suspension should be weighted considering the risk of hyperglycemia, M-ALA, and CA-AKI. Metformin represents the first-line therapy for diabetic patients [3], supported by the large body of evidence that confirmed its efficacy in providing adequate fasting glycemic control and attaining HbA1c level below 7% or 53 mmol/mol, leading to significant reduction of cardiovascular morbidity and mortality as compared with insulin or sulfonylureas [29]. Conversely, the risk of M-ALA in the overall population, in the absence of predisposing factors for metformin accumulation, is considered negligible, as confirmed by a systematic review and meta-analysis including 70,490 patient-years of metformin use and 55,451 patient-years not treated with metformin from 347 randomized and observational studies with broad inclusion criteria (143 studies enrolled patients with mild to moderate renal failure), which did not report any case of lactic acidosis [23]. Nonetheless, none of the studies included in this meta-analysis enrolled patients receiving contrast agents or undergoing coronary angiography. Indeed, there is extremely limited evidence on the risk of CA-AKI and M-ALA with metformin continuation before and after coronary angiography and PCI. A prespecified analysis from the GIPS-III trial, which randomized non-diabetic patients without renal impairment presenting with ST elevation myocardial infarction to metformin or placebo early after primary PCI [30], did not show any increase in the risk of CA-AKI with metformin [12]. Similarly, other observational and randomized studies focused on diabetic patients without severe renal impairment showed that metformin continuation before and after angiography is not associated with higher risk of CA-AKI or M-ALA as compared with metformin discontinuation [9, 31]. Most of the available evidence, however, is affected by several bias or potential confounders. First, none of these studies was powered to detect a significant difference in outcomes such as CA-AKI or M-ALA [23]. Second, key elements in the evaluation of the safety of metformin continuation were often unreported or missing in a relevant percentage of the included population. For instance, a recent trial randomized 404 diabetic patients to continue or suspend metformin before coronary angiography and PCI. The study did not find any differences in the risk of the primary endpoint, M-ALA, but—as properly acknowledged by the authors—the study was far from being adequately powered for the primary endpoint, as well as for the secondary endpoints, CA-AKI and 1-week and 6-month mortality. Of note, preprocedural lactate levels, hydration amount, and the prevalence of known risk factors for CA-AKI (e.g., anemia and reduced left ventricular ejection fraction) were not reported. Lastly, the trials planned to enrol 500 patients, but the coronavirus disease-19 pandemic forced the authors to prematurely stop the enrolment [11].

### Current practice and inconsistency in guidelines

Current clinical practice guidelines only marginally take into account the evidence provided thus far, probably reflecting the fear of M-ALA and its related mortality and the limitations of the studies evaluating the risk of metformin continuation in patients undergoing coronary angiography and PCI. In addition, guidelines are inconsistent in several aspects, such as timing to stop and restart metformin and to check creatinine and eGFR before and after the procedure [7, 32].

Our study provides evidence-based support to the current European guidelines on myocardial revascularization, which recommend checking renal function in patients assuming metformin and withhold metformin if renal function deteriorates (class of recommendation I, level of evidence C). The findings from our prospective study shows that—in case of limited risk for metformin accumulation—metformin continuation does not lead to a significant increase of lactate levels at 72 h as compared to preprocedural levels and that creatinine assessment before and at 72 h after coronary angiography and PCI ensures the safety of metformin continuation. Moreover, we observed that metformin continuation is associated with adequate glycemic control and with a low risk for CA-AKI. Based on our findings, metformin should not be discontinued prior to coronary angiography and PCI. Creatinine assessment at 72 h after the procedure assures to identify all patients with CA-AKI and subsequent risk of metformin accumulation.

The present study should be interpreted considering some limitations. First, despite the prospective collection of procedural, pharmacologic, and clinical features, our results can only be considered hypothesis-generating, and a randomized controlled trial is required for a definite confirmation. However, a randomized trial aiming at evaluating the risk of M-ALA as compared to placebo is unfeasible, considering the excessively large sample size needed. A randomized trial powered to detect differences in a surrogate marker of M-ALA, such the increase of lactate, could be the best option to definitely quantify the risk of this complication among patients assuming metformin and scheduled for coronary angiography and PCI. Second, based on the study exclusion criteria, our findings cannot be applied to high-risk patients, such as those with severely reduced ejection fraction or severe chronic kidney disease. However, most patients presenting one or more exclusion criteria are not eligible for metformin therapy, rendering our findings generalizable to the large majority of patients treated with metformin undergoing coronary angiography and PCI.

In conclusion, metformin continuation prior to coronary angiography and PCI is not associated with increased levels of lactate or decline in renal function in diabetic patients without high-risk features for its accumulation, such as severely reduced ejection fraction or severe chronic kidney disease, suggesting that metformin suspension before coronary angiography and PCI is not necessary.

## Supplementary Information

**Additional file 1: Table S1.** Number of patients enrolled in each center. **Table S2.** Risk of CA-AKI estimated by the Mehran and the Mehran 2.0 score. **Table S3.** Correlation between hydration amount and lactate levels.

## Data Availability

Data are available on reasonable request.

## Abbreviations

CA-AKI: Contrast-associated acute kidney injury
CKD: Chronic kidney disease
DM: Diabetes mellitus
eGFR: Estimated glomerular filtration rate
LVEF: Left ventricular ejection fraction
M-ALA: Metformin-associated lactic acidosis
PCI: Percutaneous coronary intervention

## Declarations

### Patient and public involvement

Patients and/or the public were not involved in the design, or conduct, or reporting, or dissemination plans of this research.
